# Study on Rolling Defects of Al-Mg Alloys with High Mg Content in Normal Rolling and Cross-Rolling Processes

**DOI:** 10.3390/ma16186260

**Published:** 2023-09-18

**Authors:** Seong-Sik Lim, Je-Pyo Hong, Minki Kim, Young-Chul Park, Sang-Mock Lee, Dae-Yeon Cho, Chang-Hee Cho

**Affiliations:** 1 Molding & Metal Forming R&D Department, Korea Institute of Industrial Technology, Incheon 21999, Republic of Korea; sslim@kitech.re.kr (S.-S.L.); mkim@kitech.re.kr (M.K.); 2 R&D Center, RtoB Co., Ltd., Incheon 21632, Republic of Korea; hjp@rtob.co.kr (J.-P.H.); ycpark@rtob.co.kr (Y.-C.P.); smlee@rtob.co.kr (S.-M.L.); cdy@rtob.co.kr (D.-Y.C.); 3 Department of Mechanical Engineering, Inha University, Incheon 22212, Republic of Korea; 4 Department of Materials Science and Engineering, Inha University, Incheon 22212, Republic of Korea; 5 Department of Materials Science and Engineering, Northwestern University, 2220 Campus Drive, Evanston, IL 60208, USA

**Keywords:** high-Mg-content Al-Mg alloy, finite element analysis (FEA), electron backscattered diffraction (EBSD), cross-rolling, defect, Goss texture

## Abstract

This study investigated defect formation and strain distribution in high-Mg-content Al-Mg alloys during normal rolling and cross-rolling processes. The finite element analysis (FEA) revealed the presence of wave defects and strain localization-induced zipper cracks in normal cold rolling, which were confirmed by the experimental results. The concentration of shear strain played a significant role in crack formation and propagation. However, the influence of wave defects was minimal in the cross-rolling process, which exhibited a relatively uniform strain distribution. Nonetheless, strain concentration at the edge and center regions led to the formation of zipper cracks and edge cracks, with more pronounced propagation observed in the experiments compared to FEA predictions. Furthermore, texture evolution was found to be a crucial factor affecting crack propagation, particularly with the development of the Goss texture component, which was observed via electron backscattered diffraction analysis at bending points. The Goss texture hindered crack propagation, while the Brass texture allowed cracks to pass through. This phenomenon was consistent with both FEA and experimental observations. To mitigate edge crack formation and propagation, potential strategies involve promoting the formation of the Goss texture at the edge through alloy and process conditions, as well as implementing intermediate annealing to alleviate stress accumulation. These measures can enhance the overall quality and reliability of Al-Mg alloys during cross-rolling processes. In summary, understanding the mechanisms of defect formation and strain distribution in Al-Mg alloys during rolling processes is crucial for optimizing their mechanical properties. The findings of this study provide insights into the challenges associated with wave defects, strain localization, and crack propagation. Future research and optimization efforts should focus on implementing strategies to minimize defects and improve the overall quality of Al-Mg alloys in industrial applications.

## 1. Introduction

Lightweighting has been dealt a significant objective, because carbon emissions should be reduced by improving the fuel efficiency of vehicles [1,2], therefore, moving away from heavy metals, such as steels, copper alloys, etc., to lightweight materials, like Al alloys, Mg alloys, and carbon-based materials. Among those substitutional candidate materials, Al-Mg alloys are recognized as the best materials, specifically in the area of body sheets for vehicles or sheets for battery packs of electric cars, because of their properties, which are high strength, good ductility, and ease of fabrication [1,2]. For these reasons, the Al-Mg alloys have been widely investigated, but there are several problems to apply to the industrial area, such as low r-value, texture control, and stress corrosion cracking [3,4,5,6,7,8,9,10]. In order to solve these problems, the addition of Mg content has been suggested by several researchers [8,9,10,11]. Mg addition is effective to control texture evolution, increase r-value, and change the grain boundary condition, which is favorable to resisting stress corrosion cracking.

Particularly, it was confirmed that Al-Mg alloys with high Mg content follow the brass-type texture evolution during a cold rolling process by Cho et al. [8,9,10,11]. According to the research, the evolution of brass-type texture was observed in Al-6Mg alloy, and it was confirmed that it was due to the restriction of cross slip and acceleration of planar slip due to the high Mg content. However, it was shown that the addition of Mg content did not contribute to strain distribution and additional mechanical property improvement due to the absence of mechanical twin formation. Furthermore, high Mg content causes the Portevin Le-Chatelier effect and negative strain rate sensitivity effect [12]. These effects make irregular deformation of Al alloys. Therefore, controlling processing conditions and designing new manufacturing methods, such as intermediate rolling, cross-rolling, etc., should be considered.

Among the investigated processes, cross-rolling has unique characteristics. According to previous studies conducted by Huh et al. [13,14], it was confirmed that the cross-rolling process is effective to randomize lattice orientation distributions, even though the annealing process is conducted after cross-rolling. Comparing between conventional cold-rolling and cross-rolling processes, cross-rolling accelerates brass-type texture evolution of aluminum alloys which is favorable for improving formability. Furthermore, because a cross-rolling process involves severe plastic deformation, high stored energy can be realized in the materials, which can be seeds for granular and small-sized grains via recrystallization in annealing states [15]. As a result, plastic anisotropy is effectively suppressed by the cross-rolling process. However, in order to be applied, rolling defects and quality should be investigated. Overall strain gradient during cross-rolling in Al-Mg alloys, which can affect irregular deformation and cracking, has not been investigated and discussed yet, and the defects which occur during cross-rolling have not been resolved by researchers.

The purpose of this study is to analyze the strain distribution and defects in normal cold-rolling and cross cold-rolling processes according to the strain mode change for an Al alloy with high Mg content and the crack formation mechanism at the edge. In this study, the edge region of cross-rolled and normal rolled specimens in an Al-6Mg alloy is investigated compared to the center region of rolled specimens and simulated through finite element analysis (FEA). Through FEA, the final shape of the rolled specimens and strain distribution in the deformation state and after deformation are estimated. Then, the defects of rolled specimens are examined using the electron backscattered diffraction (EBSD) method.

## 2. Materials and Methods

**Figure 1:**
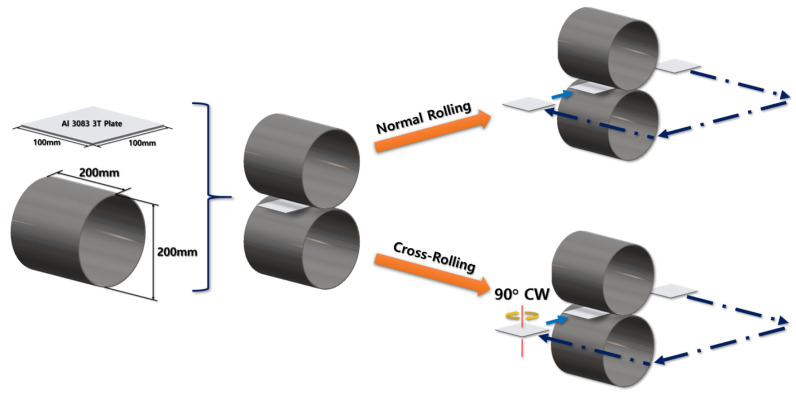
Schematic diagram of normal rolling and cross-rolling processes (Blue arrows mean the process direction).

In this study, an Al-6Mg plate with dimensions of 100 mm × 100 m × 2 mm was chosen as a virtual specimen and real test specimen. The initial grain size of cast material plate was 173 ± 21 µm. In the virtual test of FEA, the real rolling test was simulated. Two virtual rollers were set to have 200 mm in diameter and rotate at 6 rpm of angular velocity. The initial gap between the upper and lower rollers started from 2 mm and gradually decreased to 1 mm, with 0.2 mm decrement for each rolling process. Two different rolling processes were set up: normal rolling and cross-rolling. For the normal rolling process, the rolling direction was retained as the specimen moved in one direction. However, the specimen for the cross-rolling was rotated 90° clockwise when each rolling process was performed. Overall experimental procedures are illustrated in Figure 1.

In the real rolling test, the homogenized Al-6Mg alloy was used. The chemical composition of the Al-6Mg alloy was 6.0–6.4 wt% Mg and 0.048 wt% Ca, with the balance being Al. The Al billets were machined into sheet samples with 100 mm width and 2 mm thickness for the rolling process. The diameter of the upper and lower rollers was 300 mm, and the speed of the rolls was 6 rpm. To investigate the strain mode effect, each sample was cold-rolled at room temperature. Some of them were conducted through conventional normal rolling, but the others were processed via cross-rolling. In the normal cold-rolling process, the rolling direction was retained while the sample was rotated clockwise 90° with respect to the normal direction for each rolling pass in the cross cold-rolling process. In the cross-rolling process, the thickness of the sheets was reduced to 0.8 mm, corresponding to a reduction ratio of 60%. The thickness was reduced by 0.2 mm for each rolling pass.

The rolled samples were machined using electric discharge wire cutting machine (AD325L, Sodick, Yokohama, Japan). The observed plane was ground using SiC papers from #1000 to #4000 and 1 µm diamond suspension. Then, it was polished again using vibratory polisher (VibroMet, Buehler, Lake Bluff, IL, USA) with 0.02 µm colloidal silica. The microstructure of those polished specimens was investigated using the EBSD method. The EBSD data were acquired using a field-emission scanning electron microscope (FE-SEM SU5000, Hitachi, Tokyo, Japan) equipped with an EBSD camera (Velocity Super, EDAX-Ametek, Pleasanton, CA, USA). To ensure the reliability of EBSD data, the measurement region was designated to be 1000 µm × 1000 µm, and the step size was 1.0 µm. According to the collected EBSD data, inverse pole figure (IPF) maps, kernel average misorientation (KAM) data, and orientation maps were calculated and plotted through orientation imaging microscopy (OIM) program version 8.5 (TSL OIM Analysis 8.5, EDAX-Ametek, Pleasanton, CA, USA). The observed planes of the specimens were normal direction. The observed areas are the estimated areas, which have rolling defects.

## 3. Methodology: Finite Element Analysis (FEA) via Abaqus

FEA for normal rolling and cross-rolling was performed using Abaqus 2020. First, a specimen plate and a roller were modeled. Their dimensions were designed as mentioned in Section 2. Experimental Procedures: The plate and the roller were defined to be deformable and discrete rigid, respectively. Then, the material properties were set and applied to the specimen. Its specific property values are shown in Table 1, Table 2 and Table 3.

The material properties of Al-6Mg alloy relevant to density, elasticity, and damage are shown in Table 2. Johnson–Cook model parameters were used for Al-6Mg HI 16 and steel 4340 in LS-Dyna [16]. According to the Abaqus Analysis User’s Manual, the Johnson–Cook criterion is simply defined as a form of

$${\bar{\varepsilon }}_{D}^{pl}=\left[d_{1}+d_{2}\exp\left(-d_{3}\eta \right)\right]\left[1+d_{4}\ln\left(\frac{{\dot{\bar{\varepsilon }}}^{pl}}{{\dot{\varepsilon }}_{0}}\right)\right]\left(1+d_{5}\hat{\theta }\right)$$

where d_1_–d_5_ are the failure parameters, the reference strain rate, and the nondimensional temperature. Since the damage model in this study does not depend on the temperature, the nondimensional temperature is 0. Plastic properties of the alloy are tabulated in Table 2 based on Figure 12 of Laijun Wu’s work [17]. The displacement–strength curves of Al-6Mg, 6005A and 7N01 Al alloys were joints welded by Laijun Wu [17].

The following describes why the Johnson–Cook criterion was selected for this specific analysis. In Abaqus, there are various criterion models to define damage: Ductile, FLD, FLSD, Johnson–Cook, Maxe/Quade, Maxs/Quads, M-K, MSFLD, Shear, and Hashin. In this study, Johnson–Cook damage criterion was selected because the rest of the models were unavailable or inappropriate for the cold-rolling situation. FLD and FLSD criteria were for necking situation of the metal sheet. Maxe/Quade and Maxs/Quads criteria were used to predict damage initiation in cohesive elements where the cohesive layers are defined in terms of traction–separation. Shear criterion was only for shear models. Hashin damage model was not for ductile materials. Ductile, Johnson–Cook, M-K, and MSFLD damage criteria were chosen at the beginning. Among them, only Johnson–Cook damage parameters for Al-6Mg could be easily obtained.

**Figure 2:**
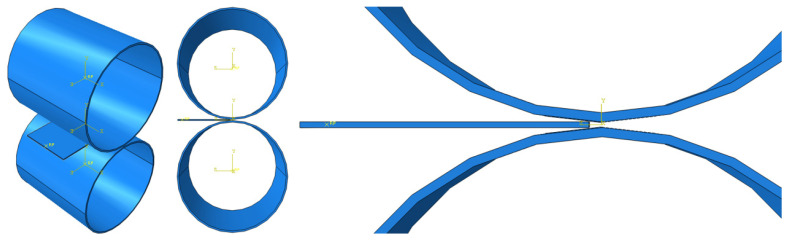
Assembly for cold-rolling FEA.

After finishing the material settings, all of the parts were properly placed in the assembly module to visualize the cold-rolling process, as shown in Figure 2. The gap between the rollers was set at 1.8 mm and reduced by 0.2 mm for every rolling process.

Each analysis underwent two *dynamic, explicit* steps. The first step was a short step finished in 0.1 s for the specimen to move close enough to the rollers. In this case, it was set to 0.03 s after several attempts with different durations. The second step was for the actual cold-rolling process. It was set to 3 s at the first rolling FEA and gradually extended up to 5 s as the specimen was stretched through the rollers. The output was recorded in 200 evenly spaced time intervals for the second step duration.

**Figure 3:**
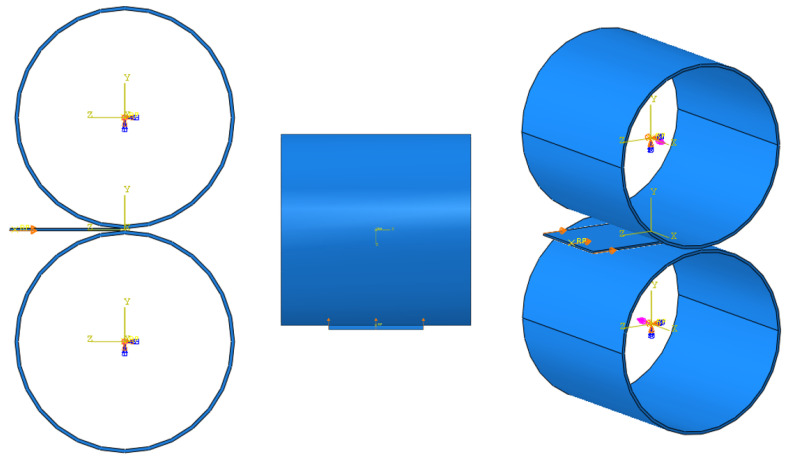
Load applied to the cold-rolling model. Blue and pink arrows mean constraints and loads respectively.

According to Figure 3, reference points and three-dimensional local coordinates were placed at the center of the rollers. For the reference points of the rollers, only rotational motion about the x-axis was allowed, and the other motions were fixed based on their local coordinates. Then, an angular velocity of 6 rpm was applied to each roller. Since the Abaqus unit system requires the angular velocity in radians per second, the velocity value was converted into 0.63 rad/s. Eventually, the upper roller and the lower roller were set to have 0.63 rad/s and −0.63 rad/s of angular velocity according to the local coordinates.

Because the operator pushed the specimen toward the gap between the rollers, a displacement of 1 mm was forced on the specimen surface where the operator pushed (Figure 3).

**Figure 4:**
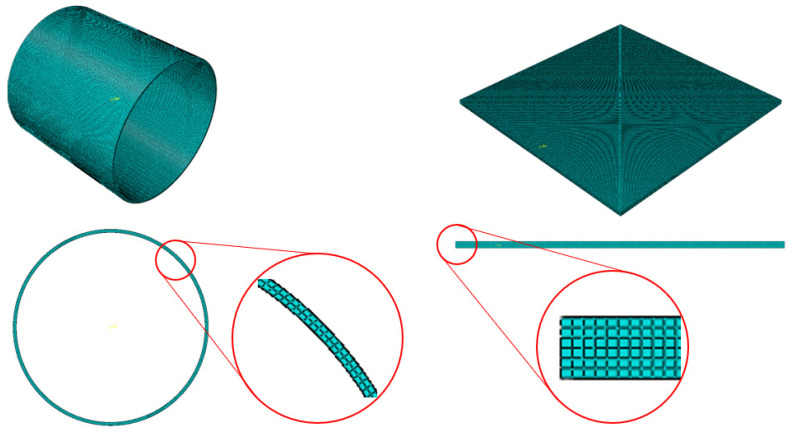
Meshed parts of the cold-rolling model.

The rollers were coarsely auto-meshed, regardless of the even distribution of the mesh using the Abaqus auto-mesher function. Since the rollers were discretely rigid parts, it does not affect the analysis result as long as their rolling surfaces are circular enough. On the other side, the specimen plate was the main deformable model to be analyzed. The 2 mm thick specimen was finely sliced into six layers so that the result has enough precision to see how the plate deforms through the rolling process. It made the layer thickness 0.333 mm along the y-axis. The number of elements along the other axis was decided so that each element had as cubic a shape as possible. Consequently, a specimen plate with 100 mm×100×2 mm dimensions was fully meshed, and its element size was 0.384 mm×0.333 mm×0.384 mm. The meshed parts are illustrated in Figure 4.

The element type of the specimen part was C3D8R, which means an 8-node linear brick, reduced integration, and hourglass control. Additionally, its element library referred to explicit, and distortion control was deactivated to see drastic dimensional change in the rolling process. In addition, element deletion was activated considering the situation that a huge crack occurs and splits the plate apart based on the Johnson–Cook damage criterion.

## 4. Results

**Figure 5:**
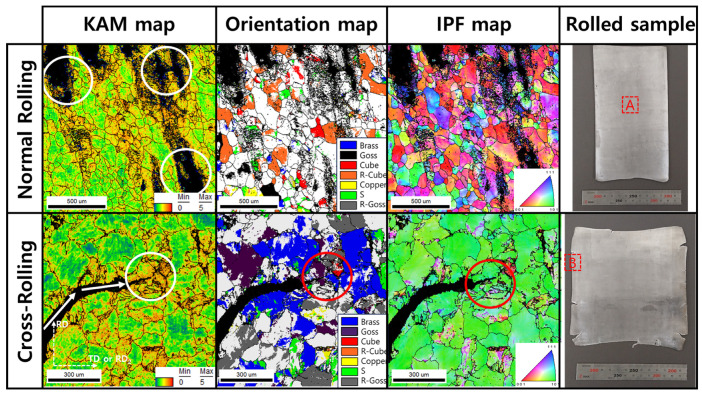
Normal rolled and cross-rolled EBSD results (KAM maps, orientation maps, IPF maps, measurement location, which is shown in rolled sample, the circles show the crack formation and its propagation).

Figure 5 shows the EBSD results of normal cold-rolled and cross-rolled specimens. The normal rolled specimen’s grain size is 152 ± 92 µm, and the cross-rolled one is 132 ± 71 µm. In this figure, particularly, this figure indicates the defects of surface and edge regions of specimens. There are defects from the rolling process, which are wavy edges, edge cracks, zipper cracks in the center of plate, and alligatoring. Among those known rolling defects, zipper cracks and edge cracks are identified in Al-Mg alloys with high Mg content. Particularly, the zipper cracks are shown after the normal cold-rolling process, but in cross-rolling, edge cracks are strongly shown. These defects are identified by the confidence index (CI) value, which is calculated in the EBSD OIM program. At the low-CI value region, there is black, but at the high value, there is not. CI values are calculated during EBSD observation based on the intensity of the Kikuchi line and eigenvalue of materials. Distortion of lattice, defects, or other factors affect the acceleration of low CI value. In this study, the formation of cracks makes the CI value lower.

According to the KAM maps in Figure 5, the region around cracks has a high value of KAM. The KAM value means the misorientation between adjacent points, which is observed via EBSD [8,11,12]. In other words, the KAM values are exhibited by the formation of geometrical necessary dislocations, which can be considered as stored energy. It can be established that high stored energy is related to forming cracks and propagation. The stored energy is also related to orientation evolution. Around cracks, there are oriented grains, such as rotated cube (R-cube) in normal rolling and Brass component in cross-rolling. The highly deformed region is oriented to ideal rolling texture component. The ideal rolling texture components are listed in Table 4.

**Figure 6:**
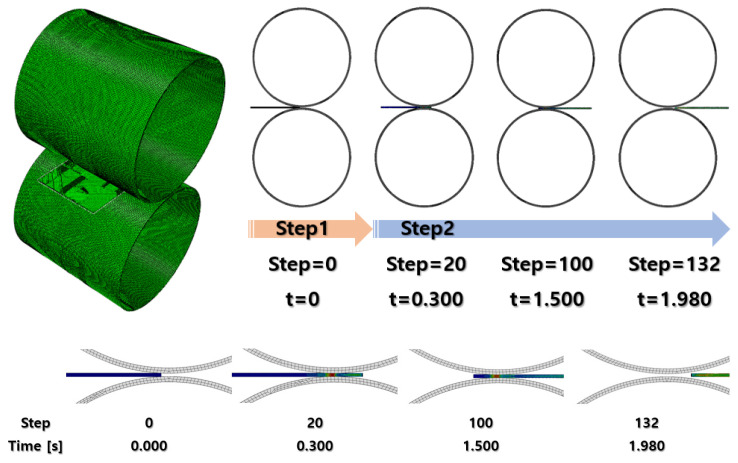
First rolling process result.

**Figure 7:**
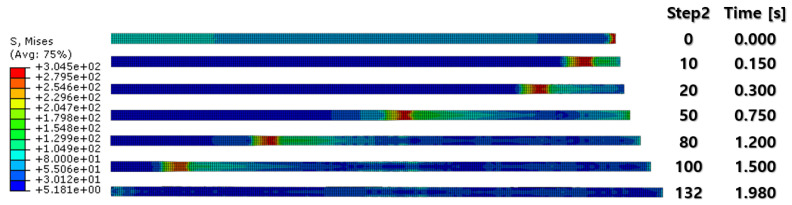
FEA result of first rolling process (side section view).

**Figure 8:**
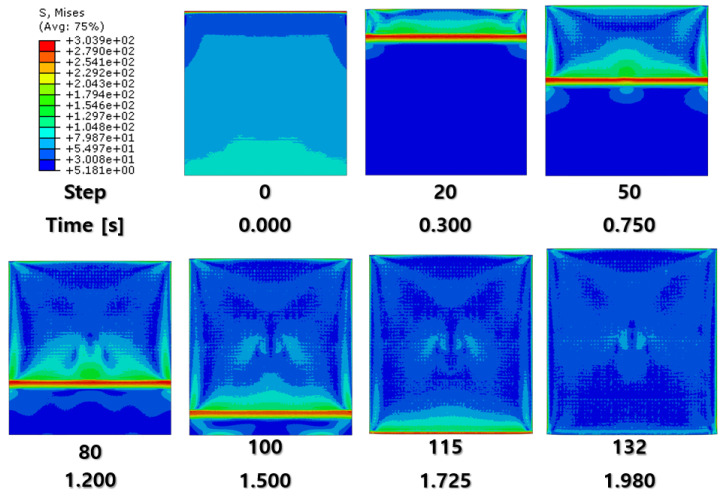
FEA result of first rolling process (top section view).

**Figure 9:**
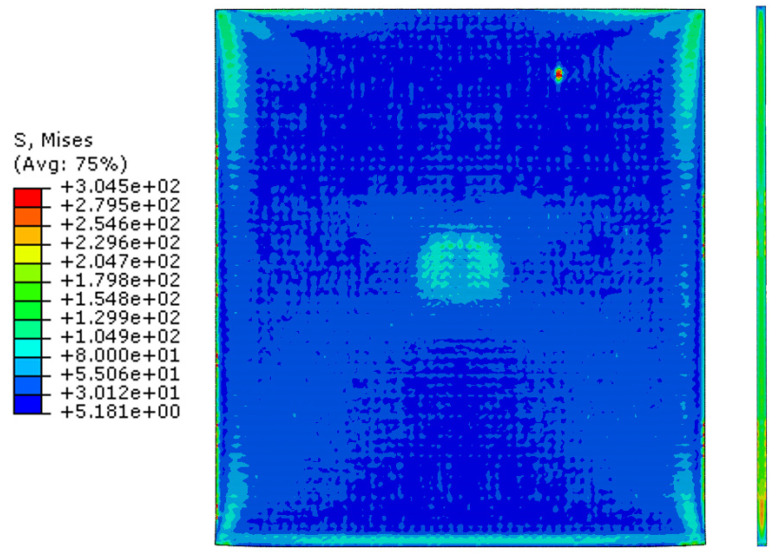
FEA result of first rolling process (external surfaces).

The FEA results of the first rolling process are illustrated below (Figure 6, Figure 7, Figure 8 and Figure 9).

Figure 7 and Figure 8 show a section view of the specimen over time under the first cold-rolling process, which machines a 2 mm thick aluminum alloy plate into a 1.8 mm thickness plate. According to the figures, as its thickness becomes thinner through the tiny gaps between the rollers, over 300 MPa pressure is applied to the plate, stretching the specimen in its transitional direction. Moreover, it is observed that the residual stress after the high pressure over its yield strength is not stabilized and has the possibility to affect the machining quality of the successive cold-rolling processes. The external shape of the cold-rolled specimen is illustrated in Figure 9. It describes the concentration of the residual stress at each edge of the specimen with light colors. Some parts are excessively deformed and expressed as red spots—top right and both side edges of the specimen in top view (Figure 9).

**Figure 10:**
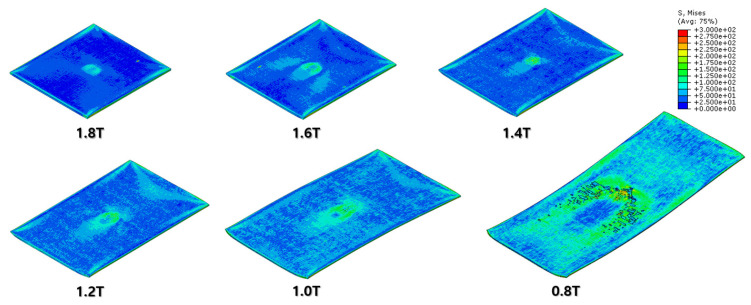
FEA result of normal cold-rolling results (isometric).

**Figure 11:**
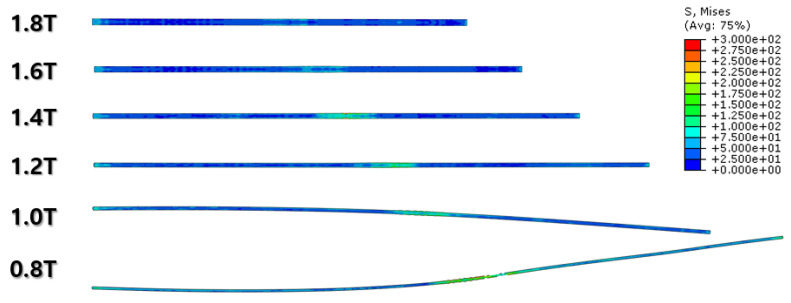
FEA result of normal cold-rolling results (side section).

**Figure 12:**
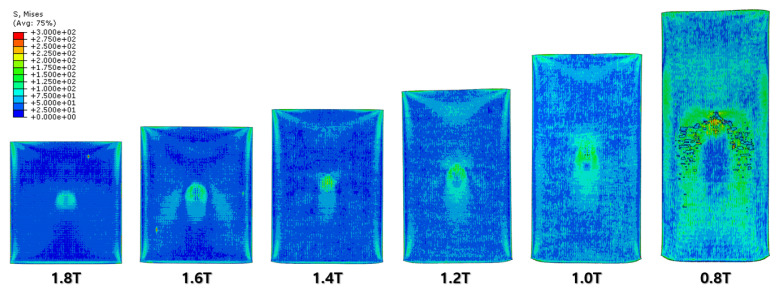
FEA result of normal cold-rolling results (top).

**Figure 13:**
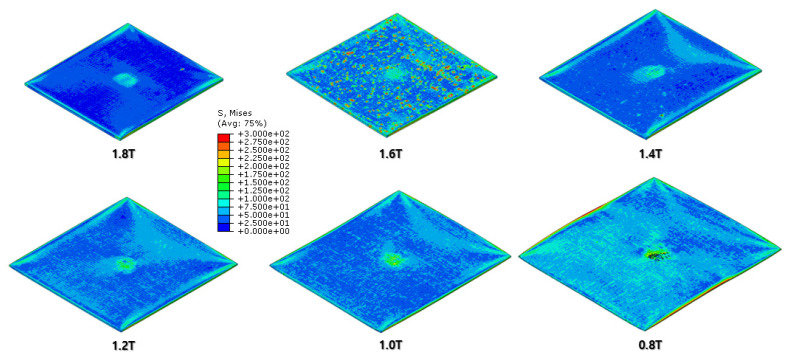
FEA result of cross cold-rolling results (isometric).

**Figure 14:**
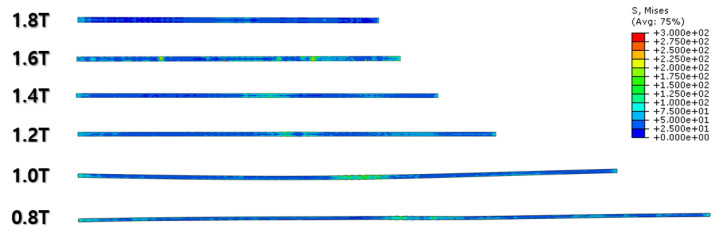
FEA result of cross cold-rolling results (side section).

**Figure 15:**
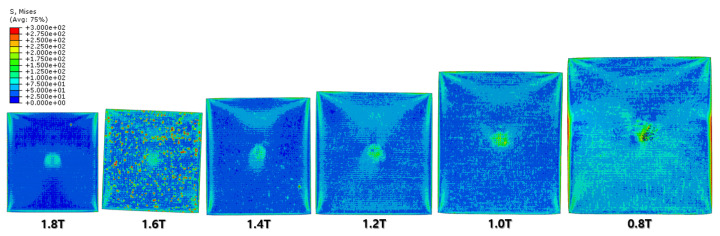
FEA result of cross cold-rolling results (top).

The specimen after the first rolling process in Figure 8 underwent the next cold-rolling processes based on Figure 1 and the explanation in Figure 2. Experimental Procedures: The thickness of the specimen was reduced by 0.8 mm, with a 0.2 mm decrement for each rolling process, whose machining results are elaborated below (Figure 10, Figure 11, Figure 12, Figure 13, Figure 14 and Figure 15). Further FEA result figures from Figure 10 to Figure 15 are scaled from 0 to 300 MPa for ease of comparison between the normal and the cross cold-rolling processes.

Figure 10, Figure 11 and Figure 12 and Figure 13, Figure 14 and Figure 15 show the FEA results of normal rolling and cross rolling, respectively. Both results are scaled in the same strength range from 000 MPa to 300 MPa.

**Figure 16:**
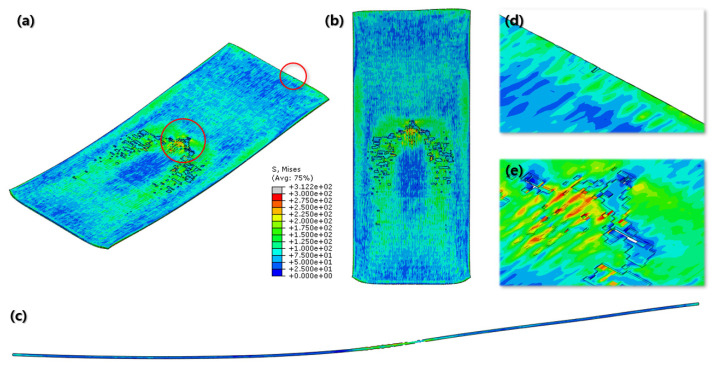
Final FEA result of normal cold-rolling process; isometric view (**a**); top view (**b**); side section view (**c**); crack at front edge (**d**); defects at center (**e**).

**Figure 17:**
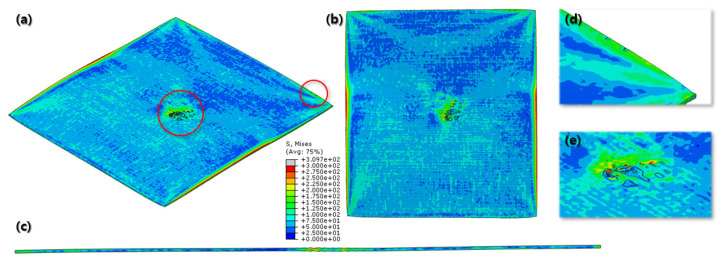
Final FEA result of cross cold-rolling process; isometric view (**a**); top view (**b**); side section view (**c**); cracks at front edge (**d**); defects at center (**e**).

According to the FEA results, the normal rolling process stretches the specimen in a single direction and, remarkably, changes the original shape of the specimen while the cross-rolling process does not due to the rotation of the rolling direction. Figure 12 and Figure 15 state that the center and side edge of the specimen in the rolling direction are under the largest pressure in the overall cold-rolling process, including both the normal and cross-rolling methods. However, larger zipper cracks and a condition of ease to make the wave defect are detected in the normal rolling process compared to cross rolling as the specimen after the normal rolling process should endure a larger moment from its weight due to its longer length than one from the cross-rolling process. In Figure 11, the normal rolled specimen showed a phenomenon of bending in a downward direction and finally in an upward direction. This analysis is divided into a dynamic step where the specimen is placed between rollers and an explicit step where rolling is performed. After rolling, the deformed specimen is moved back to the roller inlet, but it cannot be positioned exactly in the rolling direction. At this time, the moment of the specimen, which is slightly bent up or down based on the rolling direction, approaches the roller inlet end in the dynamic step and becomes stuck, the explicit step proceeds, and rolling occurs, causing pressure to be applied to the upper and lower surfaces of the specimen. The bearing stress is different. The difference in stress between the upper and lower surfaces aggravates the bending phenomenon of the specimen during the rolling process. In the actual rolling process, there are two ways to prevent the specimen from bending after rolling. First, when the operator pushes the specimen into the entrance end of the roller, the angle is adjusted based on experience to minimize the bending of the specimen. More specific figures of the final FEA results from both rolling processes are illustrated below (Figure 16 and Figure 17).

According to Figure 16 and Figure 17, both the normal and cross-rolling results show defects at the front edge and center of the specimens created by excessively large strain. As mentioned before, the normal rolling result has more defects, such as larger zipper crack and wave defect and even a hollow hole at the center of the specimen due to its higher moment from its stretched length via the rolling method. When it comes to defects at the front edge, the result from the cross-rolling process has more defects than the normal rolling result.

**Figure 18:**
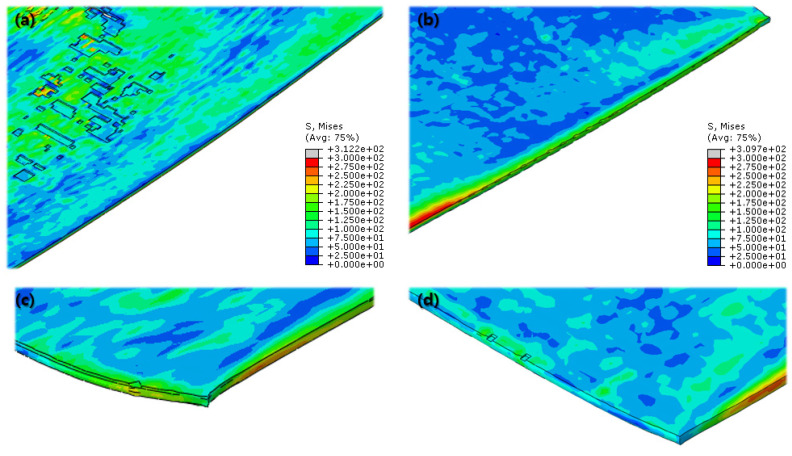
Comparison of the Final FEA results from normal and cross cold-rolling processes; side edge after normal cold-rolling process (**a**); side edge after cross cold-rolling process (**b**); end corner after normal cold-rolling process (**c**); end corner after cross cold-rolling process (**d**).

Even more details of the FEA results are described above (Figure 18). It shows the wrinkled side edge of the specimen after cross rolling, which does not appear under the normal rolling process. It is formed via the 90-degree rotation of the specimen for each rolling, which is the major difference between the normal and cross-rolling processes. The wrinkly front and rear edges of the specimen strongly strained right before cracking from the previous rolling become side edges for the next rolling process as the specimen rotates 90 degrees. The wrinkly side edges stretched along the next rolling direction become even easier to crack during next cross-rolling process as the front and rear edges containing higher residual stress than the normal rolling process. Likewise, Figure 16 and Figure 17 show that the specimen from the cross-rolling process has red side edges, which means higher stress than the one from the normal rolling process. According to (c) and (d) in Figure 18, (d) has more cracks than (c). Moreover, (c) from the normal rolling process shows a rectangular corner from its original shape, but, meanwhile, (d) from the cross-rolling process has a pinched-like corner because it was pressured over and over in the same rolling direction.

## 5. Discussion

Al-Mg alloys are widely used materials that offer a combination of lightweight properties and enhanced strength [1,2]. Among the various processing methods for Al-Mg alloys, conventional normal rolling and cross rolling are commonly employed. These two methods have a notable influence on defect formation and strain distribution during the processing of the alloy [13,14,15,20]. Understanding and controlling these defects and strain distributions are crucial as they can significantly affect the final mechanical properties of the alloy. Hence, this paper aims to investigate the strain distribution and rolling defects affected by strain localization in high-Mg-content Al-Mg alloys during both normal rolling and cross-rolling processes [21,22,23,24,25]. By doing so, we seek to propose strategies to mitigate these issues.

In the FEA conducted to predict the strain distribution, only the influence of the processing itself was considered, excluding the effects of thermal energy generation and the segregation of elements. In the case of normal cold-rolling, the formation of zipper cracks and the condition to make wave defects due to strain localization near the center were predicted, clearly indicating an uneven distribution of strain. The experimental results also support the formation of zipper cracks, and based on the results of KAM analysis and texture formation, it is inferred that crack formation and propagation are caused by the concentration of shear strains. KAM indicates that geometrically necessary dislocation exists [26], so high KAM regions mean highly dislocated piled-up regions. In Figure 16, the region of high value of strain is shown. The region was detected using EBSD, which is shown in Figure 5. In this figure, the formed zipper cracks are shown, and around these cracks, high KAM values are clearly shown. Around these cracks, grains are highly oriented rotated cube orientation components. The component is evolved by shear strain. In other words, the concentration of shear strain, which is caused by rolling, results in high stored energy, which is indicated by KAM, and texture evolution, as well as crack formation and propagation.

The wave defects observed using the FEA were observed in the actual tested specimens, albeit to a lesser extent due to the small size of the specimens, making it difficult to quantify the variations. However, if applied to actual mass production processes, it is expected that the defective rate will significantly increase due to wave defects. In particular, when the rolling process is applied to specialized shape processing rather than flat rolling, the issue of wave defects is expected to become more pronounced. In conclusion, the FEA results reveal the presence of wave defects and strain-localization-induced zipper cracks in normal cold rolling. These defects were confirmed in the experimental samples, albeit with limited quantification due to the sample size. Nevertheless, if implemented in real-world mass production processes, the detrimental effects of wave defects are expected to increase the defect rate significantly. It is especially anticipated that specialized shape processing using the rolling process will exacerbate the issue of wave defects [21]. Further research and optimization efforts are required to address these challenges and enhance the overall quality of Al-Mg alloys during rolling processes. Although the positional deviation in this study is not large in terms of the overall length, it can cause a wave defect and cause a problem when applied to the mass production process.

In contrast to normal rolling, the FEA results show a relatively uniform distribution of strain in the cross-rolling process (Figure 15). Furthermore, the influence of wave defects was predicted to be minimal. These findings were consistent with the results obtained from the actual experiments. It suggests that the nature of the cross-rolling process itself does not induce significant wave defects. However, strain concentration was observed at the edge and center regions, with a predicted formation of zipper cracks at the center. In the actual experiments, the formation of edge cracks was observed due to stress concentration at the edges. The formation of cracks in the experiments was more pronounced and exhibited more significant propagation compared to the predictions from FEA. Overall, the cross-rolling process demonstrates certain advantages over normal rolling in terms of strain distribution and the reduced influence of wave defects. However, the potential for crack formation, particularly at the center and edge regions, should not be overlooked. Further investigations and optimization strategies are necessary to address these concerns and enhance the overall quality of Al-Mg alloys during the cross-rolling process.

Upon examining the formation and propagation of cracks in the cross-rolling process, it was observed that cracks exhibited a change in direction at specific locations (Figure 5). Upon closer inspection of these crack bending points, it was evident that stress concentration played a role, typically aiding in crack propagation [27,28]. However, it appeared that the crack propagation was impeded. The reason for this hindrance can be attributed to texture evolution. Analyzing the grain orientations at the bending points revealed the development of the Goss texture component. Among the ideal rolling texture components for FCC metals, which includes orientations that form the deformation texture, Goss, Brass, Copper, and S components are present. Specifically, the orientations belonging to the α-fiber (ND//{110}) category correspond to Goss and Brass. While Brass allows cracks to pass through, Goss was found to act as an orientation component that impedes crack propagation [4,29,30,31,32]. This phenomenon was also observed in the actual experimental results.

Applying this principle, several approaches can be considered to mitigate the formation and propagation of edge cracks. Firstly, establishing alloy and process conditions that promote the formation of the Goss texture at the edge region can be a viable solution. Given that Goss orientation hinders crack propagation, it can serve as an efficient mechanism. Secondly, implementing an intermediate annealing process can alleviate the stress accumulation at the edge, effectively preventing crack formation at its origin [33,34,35].

In summary, the investigation revealed that cracks in the cross-rolling process exhibited directional changes at specific points, likely due to stress concentration. The hindrance of crack propagation was found to be associated with the development of the Goss texture at these bending points. To address edge crack formation and propagation, potential solutions involve promoting Goss texture formation through alloy and process conditions, as well as introducing intermediate annealing to alleviate stress accumulation at the edge. Further research and optimization of these approaches are essential to enhance the quality and reliability of Al-Mg alloys during cross-rolling processes.

Rolling is an essential process for the manufacturing of metal materials, but it can lead to the formation and propagation of rolling defects, which can degrade quality. The present study investigated the rolling defects in high-Mg-content Al-Mg alloys and proposed methods to minimize them. The results showed that zipper cracks formed at the center of the sheet during normal rolling, while edge cracks and zipper cracks were both observed during cross-rolling. These defects can grow as the rolling process progresses, so it is necessary to prevent their formation. One possible solution is to reduce the reduction per pass and increase the number of passes to improve strain distribution. However, this approach may not be effective in preventing defects when the total reduction is high. Another alternative is to relieve residual stresses by annealing the material during the rolling process. Annealing at temperatures below 320 °C [10,36], where recrystallization occurs, can reduce residual stresses and help to prevent the formation of defect seeds.

In future studies, it is necessary to investigate the specific implementation methods of these approaches and evaluate their effectiveness in actual production environments. Additionally, research is needed to develop more effective methods by considering other factors that contribute to defect formation.

## 6. Conclusions

This study investigated the probability of defects in high-Mg-content Al-Mg alloys during normal cold rolling and cross rolling. FEA was used to predict the defects, and EBSD analysis was used to confirm the predictions. The results showed that both normal cold rolling and cross rolling can cause defects, but the types of defects are different. Normal cold rolling can cause wave defects and zipper cracks, while cross rolling can cause edge cracks and zipper cracks. These crack formations are related to the irregular deformation energy distribution, in that the strain-localized region shows the crack formation. Therefore, the suppression of crack generation in normal and cross-rolling processes of Al-6Mg alloy seems to be possible by lowering the reduction ratio for each pass and realizing a uniform strain distribution by using iterative rolling–heating processes.

## Figures and Tables

**Table 1:** Material properties of Al-Mg alloy applied in FEA (density and elasticity).

Material	Material Properties		
	Density [$kg/m^{3}$]	Young’s Modulus [MPa]	Poisson’s Ratio
Al-6Mg	2640	70,000	0.3

**Table 2:** Material properties of Al-Mg alloy applied in FEA (plasticity).

Data	Material Properties	
	Yield Stress [MPa]	Plastic Strain
1	167	0
2	200	0.05
3	250	0.075
4	275	0.1
5	300	0.14
6	320	0.2
7	310	0.222

**Table 3:** Material properties of Al-Mg alloy applied in FEA (damage).

Johnson-Cook Damage Failure Parameters	
d1	0.0261
d2	0.263
d3	−0.349
d4	0.247
d5	16.8
Reference Strain Rate [$s^{-1}$]	1
Displacement at Failure [mm]	0.02

**Table 4:** Ideal FCC rolling texture components (the orientations are indicated by Bunge’s notation [18,19]).

Orientation	Miller Indices	$\varphi_{1}$	Φ	$\varphi_{2}$
**Cube**	{001}<100>	45	0	45
**Goss**	{110}<001>	90	90	45
**Brass**	{110}<112>	55	90	45
**Rotated Goss**	{011}<011>	0	90	45
**Rotated Cube**	{001}<110>	0/90	0	45
**Copper**	{112}<111>	90	35	45
**S**	{123}<634>	59	37	63

## Data Availability

Data available on request.
